# Absorption and Tissue Distribution of Folate Forms in Rats: Indications for Specific Folate Form Supplementation during Pregnancy

**DOI:** 10.3390/nu14122397

**Published:** 2022-06-09

**Authors:** Natasha Bobrowski-Khoury, Jeffrey M. Sequeira, Erland Arning, Teodoro Bottiglieri, Edward V. Quadros

**Affiliations:** 1 The School of Graduate Studies, SUNY Downstate Health Sciences University, Brooklyn, NY 11203, USA; natasha.bobrowski-khoury@downstate.edu; 2 Department of Medicine, SUNY Downstate Health Sciences University, Brooklyn, NY 11203, USA; jeffreymsequeira@gmail.com; 3 Center of Metabolomics, Institute of Metabolic Disease, Baylor Scott & White Research Institute, Dallas, TX 75204, USA; erland.arning@baylorhealth.edu (E.A.); teodorob@baylorhealth.edu (T.B.)

**Keywords:** folate transport, folate receptor antibodies, pregnancy, fetal uptake of folates, folate deficiency

## Abstract

Food fortification and folic acid supplementation during pregnancy have been implemented as strategies to prevent fetal malformations during pregnancy. However, with the emergence of conditions where folate metabolism and transport are disrupted, such as folate receptor alpha autoantibody (FRαAb)-induced folate deficiency, it is critical to find a folate form that is effective and safe for pharmacologic dosing for prolonged periods. Therefore, in this study, we explored the absorption and tissue distribution of folic acid (PGA), 5-methyl-tetrahydrofolate (MTHF), l-folinic acid (levofolinate), and d,l-folinic acid (Leucovorin) in adult rats. During absorption, all forms are converted to MTHF while some unconverted folate form is transported into the blood, especially PGA. The study confirms the rapid distribution of absorbed folate to the placenta and fetus. FRαAb administered, also accumulates rapidly in the placenta and blocks folate transport to the fetus and high folate concentrations are needed to circumvent or overcome the blocking of FRα. In the presence of FRαAb, both Leucovorin and levofolinate are absorbed and distributed to tissues better than the other forms. However, only 50% of the leucovorin is metabolically active whereas levofolinate is fully active and generates higher tetrahydrofolate (THF). Because levofolinate can readily incorporate into the folate cycle without needing methylenetetrahydrofolate reductase (MTHFR) and methionine synthase (MS) in the first pass and is relatively stable, it should be the folate form of choice during pregnancy, other disorders where large daily doses of folate are needed, and food fortification.

## 1. Introduction

Folic acid is an oxidized form of folate (vitamin B9) used in pharmaceutical vitamin supplements that can be chemically synthesized in the laboratory, purified from natural food sources, or from microorganisms capable of synthesizing the molecule (reviewed in Revuelta et al., 2018 [1]). 

Dietary folates derived from food sources are primarily reduced forms of folates. These can become oxidized during the cooking and processing of food to present as folic acid for intestinal absorption. During transport in the intestine, most of the dietary folate forms are converted to methylfolate (MTHF) in the folate cycle by methylenetetrahydrofolate reductase (MTHFR) and recycled by methionine synthase (MS) and dihydrofolate reductase (DHFR) [2,3]. The transport of folate across the ileal enterocyte occurs primarily via the proton-coupled folate transporter (PCFT), which along with a proton, drives the transport of folates across the apical side of the ileal enterocyte; therefore, operates at a slightly acidic pH at the site of absorption [4,5,6]. This process occurs primarily in the jejunum where folate is converted to monoglutamate [7]. The high dihydrofolate reductase (DHFR) activity and other folate pathway enzymes in the ileal enterocyte rapidly convert folates to MTHF [8]. The basolateral transport of folate is purported to occur primarily via the reduced folate carrier (RFC) and to some extent via the ATP-binding-cassette dependent transporter, the multidrug-resistance-associated protein (MRP) [9]. 

Derived from intestinal absorption, liver metabolism, and exiting from tissue cells, MTHF accounts for more than 95% of circulating folate in the blood. In blood, it exists as the monoglutamate form of MTHF because of the conjugase activity in blood plasma, while intracellular forms have 5–8 glutamic acid residues coupled via peptide bonds [6,10]. The polyglutamation of folates appears to help retain the vitamin in the cell and its participation in metabolic reactions [10].

Systemic folate deficiency leads rapidly to megaloblastic anemia, which can be readily diagnosed and treated [11,12]. Subtle folate deficiency or disruption of folate metabolism in women has been linked to subfertility, miscarriage, and pregnancy-related complications including neural tube defects (NTD) in the fetus [13,14,15,16]. Prenatal folic acid supplementation with 0.4 to 1 mg of folic acid daily has reduced the incidence of NTD by about 30–50% [17]. 

Some recent publications have suggested that unmetabolized folic acid may accumulate in blood and tissues with potential for deleterious effects [18]. Most published reports have suggested an association without connecting to metabolic pathways [19,20,21]. If such an issue exists, it may become significant with a daily intake of folic acid such as in pregnancy. However, there is no direct evidence of unmetabolized folic acid affecting folate absorption, transport, or intracellular folate pathways [22,23]. Moderate folic acid supplementation in mice has produced behavioral deficits with a decrease in MTHFR pseudogene due to elevated folic acid [24]. The relevance of this observation, if any, to alteration in folate metabolism remains to be defined. Food fortification with folic acid in many countries has raised the folate status in the population and decreased the prevalence of pregnancy-related complications, but neurodevelopmental disorders persist. 

The identification of folate receptor alpha autoantibodies (FRαAuAb) in women with NTD pregnancy prompted us to re-evaluate folate supplementation during pregnancy to determine if a specific form of folate would be preferable; especially since a significant number of women (60–70%) with a history of NTD pregnancy [16] or autism spectrum disorder (ASD) in the offspring [25] are positive for the FRαAuAb [26,27]. These antibodies can prevent folate transport by blocking (they block the binding of folate to the folate receptor) or binding (they bind to the folate receptor antigen at an epitope distant from the folate binding site) [28,29]. Intervention would require pharmacologic doses of the vitamin to overcome the blocking of folate transport and correct the folate deficiency in the fetus and neonatal brain [30]. 

This study was conducted to identify the form of folate most likely to be absorbed, distributed to tissues, and efficiently transported across the placenta to the fetus and across the blood-brain barrier of the fetal brain. We have used the rat model of exposure to FRαAb established in our laboratory [31,32] to conduct these studies with the objective of identifying the form of folate best absorbed and distributed to the fetus and to the fetal brain even in the presence of FRαAb.

## 2. Materials and Methods

### 2.1. Folate Forms and Administration to Adult Rats

Folic acid (PGA, Bayer AG, Dramstadt, Germany) was solubilized as the sodium salt by suspending the powder in water and then adding 0.1 M NaOH until dissolved. An aliquot of this solution was further diluted in 0.1 M NaPO_4_ buffer (pH 7.4) to determine the exact concentration and further diluted in phosphate buffered saline (PBS) for use. L-5-methyfolate (MTHF) (Cerbios-Pharma SA, Lugano, Switzerland), the acid form was also solubilized as sodium salt as described above; d,l-folinic acid as calcium salt (Leucovorin, Spectrum Pharma, Irvine, CA, USA), and l-folinic acid as calcium salt (Levofolinate, Fusilev, Spectrum Pharma, Irvine, CA, USA) were dissolved in PBS at a concentration of 2 mg/mL, adjusted to pH 7.4. 

### 2.2. Time Course of Folate Absorption

Folate absorption was studied in adult male rats (200–250 g) because these were readily available. For each timepoint and folate form, 2 rats were used for a total of 32 rats in the absorption study. All compounds were administered by oral gavage in a volume of 0.5 mL. The dose administered was 4 mg/kg to rats fasted overnight. The rats were euthanized by CO_2_ asphyxiation at time points ranging from 30 min to 4 h, and blood was collected by cardiac puncture using a 1 mL tuberculin syringe, and transferred to a 2 mL microfuge tube to clot. The serum was separated by centrifugation and diluted with an equal volume of 0.1 M NaPO_4_ (pH 7.4) buffer containing 4% sodium (Na) ascorbate before storing the sample at −20 °C.

### 2.3. MTHF Concentration in Tissues

For measuring MTHF levels in tissues 24 h after oral administration of folate forms, we used 10 timed-pregnant GD14 Long Evans rats and 8 non-pregnant Long Evans female rats (Charles River Labs, Wilmington, MA, USA). For PGA and MTHF, two rats were used and for d,l-folinic acid and levofolinate three rats were used. Two of the GD14 rats that were not administered any oral folate forms served as controls for endogenous levels of MTHF. All rats within each experimental condition were euthanized 24 h post folate form administration and blood, liver, kidney, ovary, brain, placentas, and embryos (in pregnant rats) were collected. Blood was collected in the same manner as in the time course absorption study. Tissues were homogenized in 0.1 M NaPO_4_ (pH 7.4) buffer containing 2% Na ascorbate and kept at −20 °C. 

To determine the effect of anti-rat FRα specific antibody (FRαAb) produced in rabbits on orally administered folate forms, 8 GD14 pregnant dams were injected intraperitoneally (IP) with two doses of purified rabbit IgG fraction (total 9.5 mg; 4.75 mg each dose) each containing 100 µg (200 µg in total) of FRαAb or equivalent amount of normal rabbit IgG (NR-IgG) plus 0.5 mL normal rat serum as additional carrier protein at time zero and after 16 h. Folate forms were administered orally (1 mg each) 6 h after the second dose and the rats were euthanized 24 h after administering folate forms. Tissues were collected and processed in the same manner as the pregnant and non-pregnant rats given folate forms only. 

All rats used in these studies had free access to water and food with a normal diet containing 2 mg folic acid per kg chow as recommended by the American Institute of Nutrition (1977) but were deprived of food overnight prior to use in the studies. The rats were maintained at 22 °C and on a 12 h light/dark cycle and the experimental protocols were approved by the Animal Care and Use Committee of the State University of New York, Downstate Health Sciences University, Brooklyn, New York, USA.

### 2.4. Serum Folate Forms Determination by LC-ESI-MS/MS

Folate forms in serum samples were determined by liquid chromatography-electrospray ionization mass spectrometry (LC-ESI-MS/MS). Analysis of serum for MTHF, PGA, tetrahydrofolate (THF), formyl-THF, was performed on a Sciex 5500QTRAP mass spectrometer (Foster City, CA, USA) coupled to a Shimadzu ultra-high pressure Nexera chromatograph system (Kyoto, Japan). The MS/MS experiments were performed under positive electrospray ionization (+ESI) with multiple-reaction monitoring (MRM) using a Turbolon Spray electrospray source operating at a voltage of 5.5 kV and desolvation temperature of 500 °C. The selected precursor and fragment ions used for the measurement of unlabeled and labeled folates are summarized in Table S1 (see Supplemental Data, Table S1). The calibration curve was prepared in water containing 2 mg/mL Na ascorbate over a range of 12.5–400 nmol/L for each folate analyte. Sample preparation involved combining 50 µL of blank, standards (MTHF, PGA, THF and Formyl-THF), control, or sample with 10 µL of 62.5 mM dithiothreitol, 20 mg/mL Na ascorbate, and 2.5 µM 13-C labeled stable isotope internal standards (13C5-5-MTHF, 13C5-PGA, 13C-Formyl-THF) in 1.5 mL microfuge tubes, mixed, and incubated for 10 min at room temperature in the dark. Next, samples were deproteinized with 5 volumes of methanol containing 2 mg/mL of ascorbate, mixed, and centrifuged at 14,800 rpm (21,100× *g*) for 10 min. The supernatant was transferred to a 96-well microtiter plate for analysis. The samples were analyzed following injection of 10 µL of extract on a Synergi Hydro 4 µm × 150 × 3 mm with a 4 × 2 mm Guard column (Phenomenex, Torrance, CA, USA) maintained at 40 °C, and eluted in a gradient with buffer A (100% water with 0.1% formic acid) and buffer B (100% Methanol with 0.1% formic acid). The flow rate was 0.5 mL/min, with a gradient over a total run time of 6 min: 0.0–2.0 min, 0–100% B; 2.5 min, 100% B; 2.6–6.0 min, 0% B. Eluent flow from the column was diverted to waste at the beginning and end of each run and was only directed to the source for the period from 1.5–3.5 min. The LC-MS/MS data were acquired and processed using Analyst 1.5.2 software (Sciex, Framingham, MA, USA).

Folate standards (MTHF, PGA, THF, and Formyl-THF) and 13C-labeled stable isotope internal standards (13C5-5-MTHF, 13C5-PGA, and 13C-Formyl-THF) were obtained from Schircks Laboratories (Bauma, Zürich, Switzerland). Formic acid, ascorbate, and dithiothreitol (DTT) were obtained from Fluka and Optima, and LC-MS grade methanol from Fisher Scientific (Waltham, MA, USA). Stock standard for each folate metabolite and internal standard was prepared as 1 mmol/L solution in water containing 2 mg/mL Na ascorbate and stored at −80 °C. 

### 2.5. Serum and Tissue MTHF Quantification by Sequential Binding Radio-Assay

The procedure described above was only used to determine folate forms in serum during absorption of orally administered folates where it was critical to identify the unconverted and reduced forms of folate. Because of the methodologic limitations and complexities of assaying folate forms in tissues by the above procedure, we measured serum and tissue MTHF levels in non-pregnant and GD14 pregnant rats 24 h post-oral administration of various folate forms by a sequential binding radio-assay [33]. This time frame was chosen to study tissue distribution of orally administered folate forms because most of the folate forms are converted to MTHF during this period. Therefore, MTHF concentration provides a reasonable estimate of folate concentration in the tissue. For measuring MTHF concentration, an aliquot of tissue homogenate was diluted in 3 volumes of 2% Na ascorbate containing buffer in a glass tube, sealed with parafilm and foil, and placed in a boiling water bath for 10 min. The sample was cooled and centrifuged at 3000 rpm (4200× *g*) for 5 min. The clear supernatant was transferred to another tube and assayed for MTHF concentration by a sequential binding assay using bovine milk folate receptor alpha (FRα) as the binding protein, ^3^H-folic acid as the tracer, and MTHF as the standard [34,35] (see Supplemental Data, Figure S1).

Serum and tissue extracts were prepared fresh for the assay and any extract remaining was discarded. The assay as modified can provide a quantitative estimate of MTHF in tissue extracts. Other reduced forms of folate such as THF, methylene-THF, methenyl-THF, and formyl-THF cannot be measured in this assay because they have low affinity for the FRα used as the binding protein. However, any presence of folic acid in the tissue is likely to provide an overestimate of folate in the sample since it has a higher affinity for the FRα protein used in the assay. 

### 2.6. Statistical Analysis of Tissue MTHF Values 

Our objective was to determine which folate form is most efficiently transported to the fetus. Therefore, our most relevant quantitative comparison is between the placenta and embryo. Placenta and embryos from all pregnant rats in the study were treated as independent samples to determine if significant differences are seen in MTHF uptake with a given folate form and in the presence of FRαAb. We sampled at least 3 embryos and their corresponding placentas. In the embryonic tissues where only folate form is given, the sample size was 8, 4, 10, 19 for folic acid, 5MTHF, levofolinate and d,l-folinic acid, respectively. In embryonic tissues where NR-IgG is given, folic acid, 5MTHF, and levofolinate sample size was 4 while d,l-folinic acid was 5. The sample sizes for embryonic tissues where FRαAb was given, was 4, 3, 5, 5 for folic acid, 5MTHF, levofolinate and d,l-folinic acid, respectively.

All statistical analyses were conducted with jamovi software (https://www.jamovi.org (accessed on 30 April 2022), Sydney, Australia). A one-way ANOVA, Welch’s test was conducted to compare the accumulation of MTHF in gestational tissues (placenta and embryos) when various folate forms were administered. A post-hoc Tukey’s test determined the significant differences between the folate form administered and embryonic MTHF concentration. Placenta MTHF values between folate form groups were analyzed post-hoc by the Games-Howell test because the Shapiro-Wilk test for normality showed a *p*-value less than 0.06. To compare the effect of NR-IgG or FRαAb, a one-way ANOVA was conducted with a post-hoc Tukey’s test or Games-Howell test depending on verification from the Shapiro–Wilk test.

### 2.7. ^3^H-PGA Distribution in the Presence of FRαAb

Tissue distribution of ^3^H-PGA and the effect of FRα antibody on this distribution was determined in a GD 14 pregnant rat. Control non-immune rabbit IgG (NR-IgG) (9.5 mg) or rat FRα antibody (FRαAb-IgG) (200 µg) containing rabbit IgG (9.5 mg total) plus 0.5 mL normal rat serum was administered IP at time zero followed by 5 µCi of ^3^H-PGA (equivalent to 231 ng of PGA) IP 24 h after IgG administration. The rats were euthanized after 24 h, tissues were collected, homogenized in 1N HCl (up to 1 mL), and an aliquot was used to measure ^3^H radioactivity. 

### 2.8. B-PGA Distribution in GD14 Placenta and Embryo

Tissue distribution of folic acid was also studied by IP administration of biotin-PEGlyated on the glutamate portion of folic acid (B-PGA; 25 µg in 500 µL of normal rat serum) (Nanocs, M.W. 3400, New York, NY, USA) to GD 14 pregnant rats. The rats were euthanized at various time points from 30 min to 4 h. Their tissues were collected, fixed in lab-made Carnoy’s solution, and processed for paraffin embedding. For immunohistochemical localization of B-PGA we used the avidin/biotin/peroxidase complex (Vectastain ABC R.T.U. system, Vector Labs) followed by color development with 3,3′-diaminobenzidine (DAB) substrate (Vector Labs, Newark, CA, USA).

### 2.9. FRαAb Localization in GD14 Placenta and Embryo 

Tissue distribution of FRαAb in a GD14 pregnant rat was determined by injecting FRαAb-IgG (9.5 mg of the purified IgG fraction containing 200 µg of FRα antibody) mixed with 0.5 mL rat serum IP. The rat received one dose of the antibody at time zero and the second dose at 16 h. The dam was euthanized 6 h after the second dose. The tissues and fetuses along with the placenta were collected, fixed in Carnoy’s solution, paraffin-embedded, and processed for histologic examination. A pregnant rat injected with an identical dose of NR-IgG served as a negative control for the immunostaining of the FRαAb distribution (see Supplemental Data Figure S2). Sections 6–7 µm thick were mounted on glass slides, deparaffinized, and incubated with normal goat serum for 1 h to block goat IgG secondary antibody from nonspecifically sticking to the tissue. The sections were then incubated with 1:500 dilution of biotin-conjugated goat anti rabbit IgG secondary antibody (Vector Labs) followed by Vectastain ABC R.T.U. system (Vector Labs) and color development with DAB substrate (Vector Labs).

## 3. Results

### 3.1. Time Course of Folate Absorption

**Figure 1:**
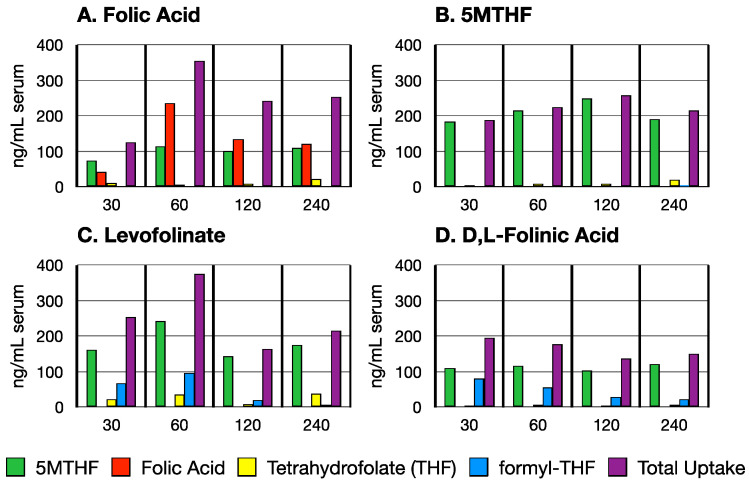
Serum concentration of folate forms following oral administration of PGA (**A**); L-5-methylfolate (**B**); Fusilev (**C**); Leucovorin (**D**); determined at 30 min, 60 min, 120 min, and 240 min post oral gavage. Values shown are ng/mL serum for all folate forms; Two rats were used for each folate form administered at each time point.

The serum concentration of orally administered folate forms was studied in adult male rats (200–250 g) because these were readily available and the objective here was to study the time course of intestinal absorption by monitoring the concentration of folates in the serum. Following oral administration of folate forms (4 mg/kg), peak folate concentration in the serum was reached by 1 h, irrespective of the form administered (Figure 1). A substantial amount of folate was retained in the serum even after 4 h, for all forms of folate.

**Figure 2:**
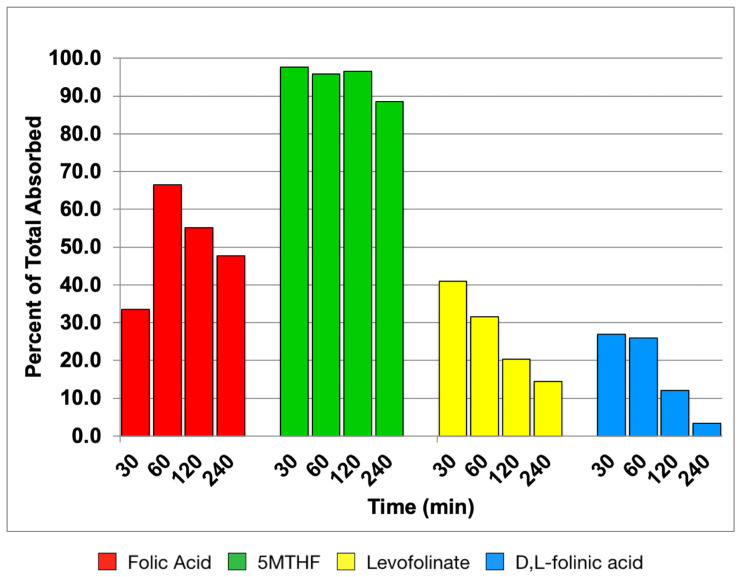
Absorbed unconverted folate form in blood. The values are expressed as a percentage of total folate in the serum. Levofolinate and d,l-folinic acid identified as unconverted are deduced from the total concentration in the serum. Therefore, the MTHF formed is likely to include THF and formyl-THF formed, which are minor components of the unconverted folate forms. MTHF administered appears to be absorbed as such without further conversion.

The three forms of folate namely levofolinate, d,l-folinic acid, and folic acid were rapidly converted to MTHF. Less than 25% of the levofolinate and d,l-folinic acid were seen in the blood at 30 min and 60 min, and approximately 50% of the folic acid remained in the native form during the 240 min of absorption, tissue distribution, and excretion (Figure 1A). Most of the MTHF remained in its native form during and following absorption at 4 h (Figure 1B). Higher total folate concentration was reached in the serum with levofolinate and folic acid (Figure 1A,C) than with d,l-folinic acid or MTHF (Figure 1B,D) but d,l-folinic acid and levofolinate appear to be more readily converted to MTHF than folic acid (Figure 2). Overall, levofolinate appeared to be somewhat better absorbed and generated higher THF over the 4 h time frame examined. 

Initially, some formyl-THF was seen with levofolinate and d,l-folinic acid and likely represents unconverted forms but by 4 h, most of this was converted to MTHF. No folic acid was formed from any of the other forms absorbed. In the time frame studied, no formyl-THF was generated from folic acid but some THF did appear by 4 h. More THF accumulated in the serum from levofolinate than from d,l-folinic acid. 

Figure 2 shows the percentage of unconverted forms in the serum for the four forms of folate at 30 min, 1 h, 2 h, and 4 h. Unconverted folic acid accounts for more than 60% of the absorbed compound at peak and only decreases to about 50% by 4 h. MTHF on the other hand, accounts for ~90% of the serum folate throughout the 4 h period. After reaching peak concentration at 30 min, d,l-folinic acid and levofolinate decrease substantially by 4 h.

### 3.2. MTHF Concentration in Non-Pregnant and Pregnant Rat Tissues Post Folate Form Administration

**Figure 3:**
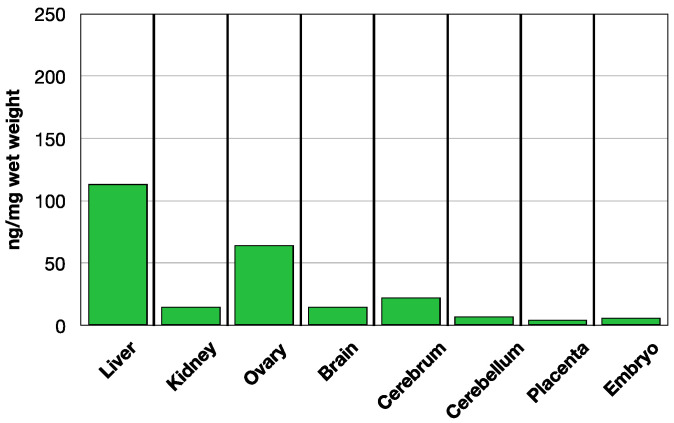
Endogenous tissue MTHF in a GD 14 pregnant dam (*n* = 2).

In the pregnant rat on normal folate replete chow (Lab Diet 5001, St. Louis, MO, USA), the baseline MTHF level is highest in the liver and was similar to that in the non-pregnant and young rats [36,37]. The ovary contained the next highest level of MTHF with lower levels in the brain. The placentas and the embryos contained substantially low levels of MTHF in comparison (Figure 3).

**Figure 4:**
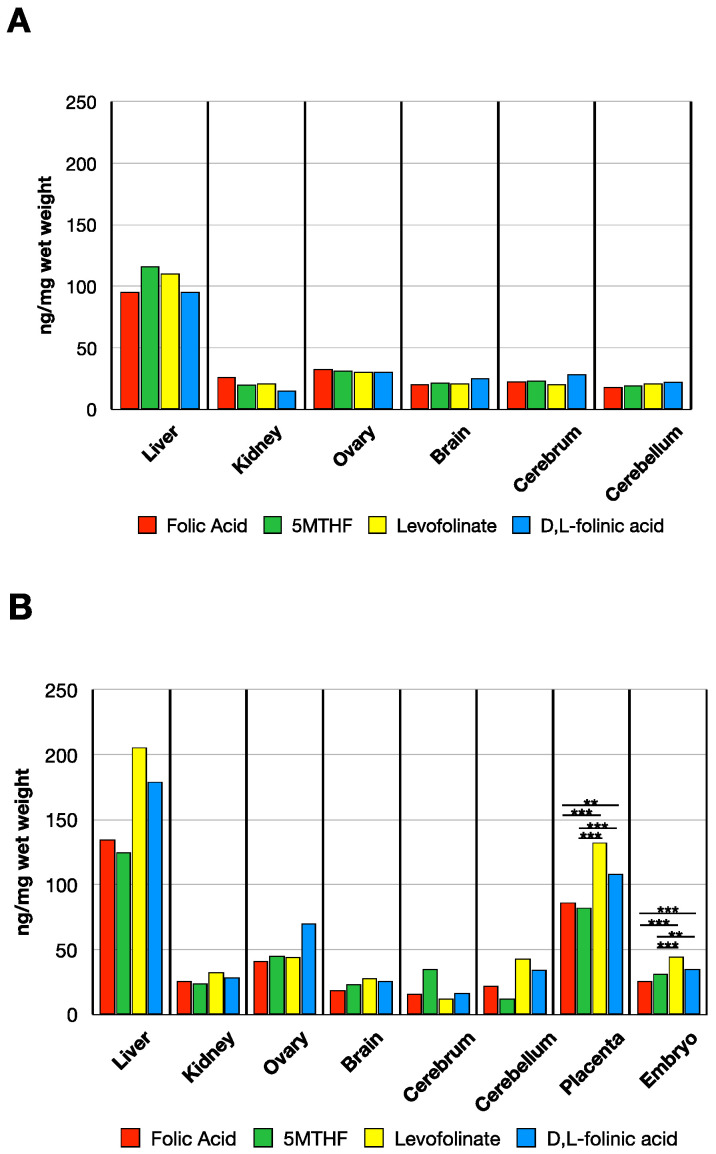
MTHF concentration in tissues of non-pregnant rats (**A**) and in GD 14 pregnant rats (**B**) 24 h post oral administration of various folate forms. (Sample sizes non-pregnant (**A**): Folic acid (*n* = 2), 5MTHF (*n* = 2), Levofolinate (*n* = 2), d,l-folinic acid (*n* = 2); Sample sizes pregnant (**B**): Folic acid (*n* = 2), 5MTHF (*n* = 2), Levofolinate (*n* = 3), d,l-folinic acid (*n* = 3)). Asterisks indicate the following: ** *p* < 0.01, *** *p* < 0.001.

When analyzing non-pregnant rats 24 h post folate administration, we observed a slight increase in liver MTHF compared to the baseline value. Liver MTHF was similar with all four forms administered. The MTHF accumulation in the kidneys, ovary, and brain was also similar with all four forms of folate administered (Figure 4A) with values comparable to baseline values in control dams shown in Figure 3. 

In GD 14 dams, tissue distribution of MTHF concentration 24 h post dosing was somewhat higher compared to non-pregnant rats. Moreover, substantially higher amount of MTHF was observed in the placentas and embryos with all forms of folate administered (Figure 3 and Figure 4b for comparison) (placenta: F(4, 14.1) = 623, *p* < 0.001, embryo: F(4, 15.4) = 146, *p* < 0.001). When comparing the MTHF concentration in placenta among the administered folate forms via Gammell–Howell test, levofolinate had higher MTHF levels than folic acid (*t*-value = −6.35, df = 12.61, *p* < 0.001) and 5MTHF (*t*-value = −7.35, df = 10.34, *p* < 0.001). Placental MTHF concentration with d,l-folinic acid was higher than with folic acid (*t*-value = −4.11, df = 24.9, *p* = 0.003) and 5MTHF (*t*-value = −5.4, df = 21, *p* < 0.001) but not when compared to levofolinate (*t*-value = 2.86, df = 18.1, *p* = 0.069). MTHF uptake in embryonic tissue was also increased when comparing levofolinate to folic acid (*t*-value = −9.34, df = 41, *p* < 0.001) and 5MTHF (*t*-value = −5.11 df = 41, *p* < 0.001) via post-hoc Tukey’s test. Moreover, d,l-folinic acid had higher concentration levels than folic acid (*t*-value = −8.3, df = 41, *p* < 0.001) and 5MTHF (*t*-value = −3.79, df = 41, *p* = 0.004), but no difference in concentration when compared to levofolinate (*t*-value = 2.4, df = 41, *p* = 0.137).

### 3.3. MTHF Concentration Post Administration of Folate Forms in the Presence of NR-IgG or FRαAb

**Figure 5:**
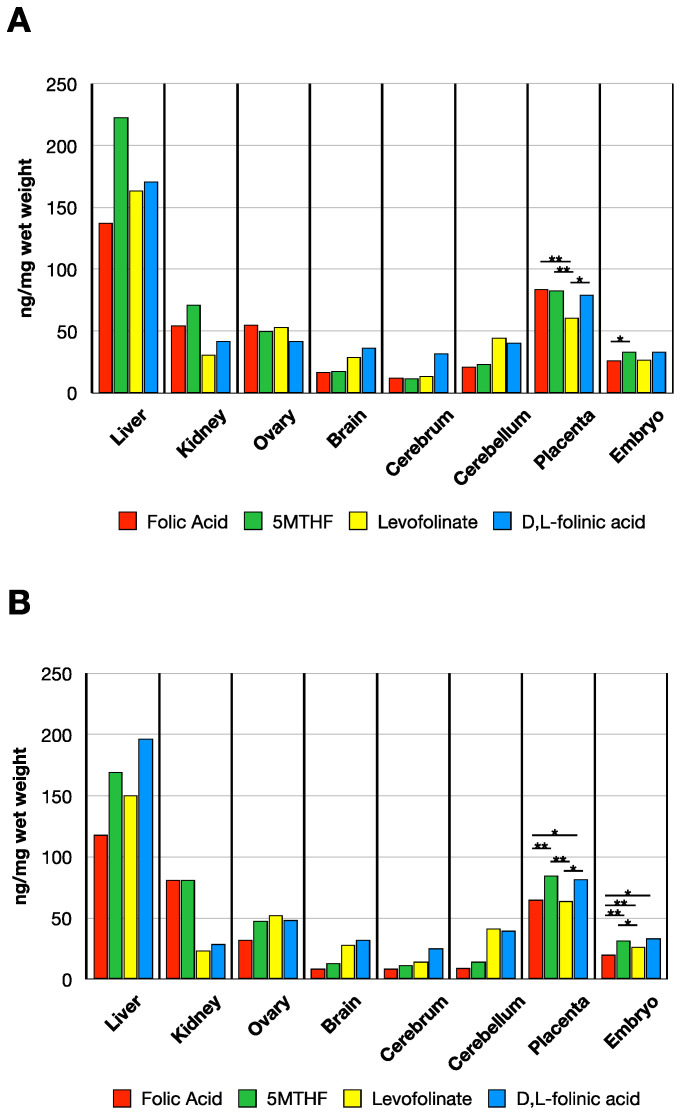
Methylfolate levels in a GD 14 pregnant rat following oral administration of folate forms, and IP administration with normal rabbit IgG (NR-IgG; (**A**)) and rabbit anti rat FRα antibody (FRαAb; (**B**)) Asterisks indicate the following: * *p* < 0.05, ** *p* < 0.01.

In dams given 2 doses of NR-IgG or FRαAb-IgG prior to oral dosing with folate forms, there was no major difference in folate distribution between NR-IgG or FRαAb-IgG administered rats suggesting that at the pharmacologic doses administered, all forms of folate are taken up and distributed to tissues including the placenta and the embryo. This distribution is not affected by the presence of FRαAb (Figure 5A,B). 

A one-way ANOVA comparing MTHF concentrations in placenta and embryo after two doses of NR-IgG showed differences among the four folate forms (placenta F(3, 7) = 9.58, *p* = 0.007; embryo: F(3, 7.09) = 17.91, *p* = 0.001). A post-hoc Tukey’s test conducted on placenta 5MTHF values for each given folate form suggested that levofolinate gave reduced concentration when compared to folic acid (*t*-value = 4.26, df = 13.0, *p* = 0.004), 5MTHF (*t*-value = 4.29, df = 13, *p* = 0.004), and d,l-folinic acid (*t*-value = −3.574, df = 13, *p* = 0.016). There are no differences between folic acid and 5MTHF (*t*-value = −0.0279, df = 13, *p* = 1.0), folic acid and d,l-folinic acid (t-value = 0.920, df = 13, *p* = 0.795), and 5MTHF and d,l-folinic acid (*t*-value = 0.949, df = 13, *p* = 0.779). The only difference in embryo MTHF concentration was observed after post-hoc Tukey’s test is between folic acid and 5MTHF (*t*-value = −3.26, df = 13, *p* = 0.028). 

Comparing folate forms after two doses of FRαAb showed MTHF decreases in placenta and embryo (Figure 5B) (placenta: F(3, 6.49) = 34.9, *p* < 0.001, embryo: F(3, 6.55) = 58.4, *p* < 0.001). After post-hoc Games-Howell test, differences between folic acid and 5MTHF (*t*-value = −8.59, df = 4.74, *p* = 0.002), folic acid and d,l-folinic acid (*t*-value = −4.518, df = 5.67, *p* = 0.018), 5MTHF and levofolinate (*t*-value = 10.127, df = 4.17, *p* = 0.002), and levofolinate and d,l-folinic acid (*t*-value = −5.004, df = 5.07, *p* = 0.015) in placental MTHF concentration were observed. There were no differences observed between folic acid and levofolinate (*t*-value = 0.520, df = 5.99, *p* = 0.951) and 5MTHF and d,l-folinic acid (*t*-value = 0.846, df = 5.55, *p* = 0.831). A post-hoc Tukey’s test to compare the embryo MTHF concentrations between the folate form groups after two doses of FRαAb shows decreases between folic acid and all reduced forms of folate (folic acid vs. 5MTHF: *t*-value = −5.27, df = 13, *p* < 0.001, folic acid vs. levofolinate: *t*-value = −3.24, df = 13, *p* = 0.029, folic acid vs. d,l-folinic acid: *t*-value = −6.598, df = 13, *p* < 0.001) as well as difference between levofolinate and d,l-folinic acid (*t*-value = −3.566, df = 13, *p* = 0.016). There were no differences between 5MTHF and both levolfolinate (*t*-value = 2.54, df = 13, *p* = 0.1) and d,l-folinic acid (*t*-value = −0.550, df = 13, *p* = 0.945). 

### 3.4. ^3^H-PGA Uptake at GD14 in the Presence of FRαAb

**Figure 6:**
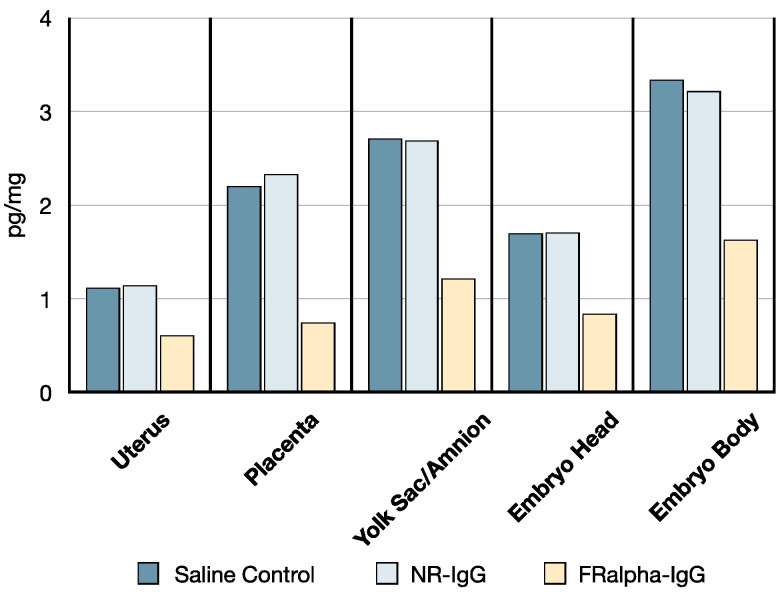
Effect of FRα antibodies on tissue distribution of ^3^H-PGA in GD 14 dams following IP injection of antibody and ^3^H-PGA. The sample sizes for the placenta and embryo in the saline, control NR-IgG, and FRαAb groups were *n* = 3, *n* = 2, and *n* = 2, respectively.

The distribution of ^3^H-PGA in tissues was similar in saline and NR-IgG administered rats. However, there was a 50% decrease in ^3^H-PGA in reproductive tissues including the placenta and embryo in the dam administered FRαAb (Figure 6). 

### 3.5. B-PGA Distribution in GD14 Tissues

**Figure 7:**
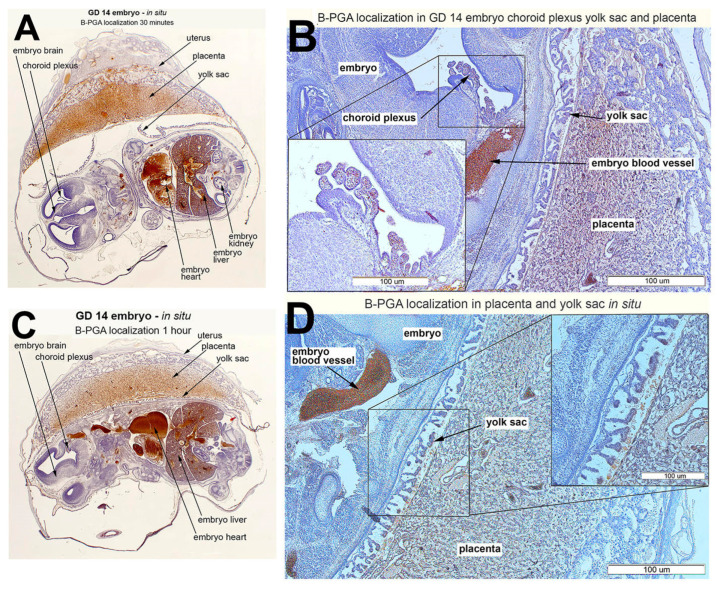
Distribution of biotin-conjugated folic acid (B-PGA) (brown staining) in the GD 14 placenta and embryo following IP injection of 25 µg of the compound. The rats were euthanized 30 (**A**) and 60 (**B**–**D**) minutes after B-PGA administration. Hematoxylin was used as the counterstain (blue/purple).

Biotin-conjugated folic acid (B-PGA) administered IP was rapidly distributed to the placenta and the embryo with more accumulation in the placenta at 30 min and more of the B-PGA transfer to the embryo at 1 h (Figure 7). The decrease in placental accumulation was reflected by an increase in accumulation in the embryo. Overall, B-PGA was distributed throughout the reproductive system including the fetal tissues and the choroid plexus. There were no observed differences in B-PGA distribution between 1 h and 4 h (data not shown). 

### 3.6. FRαAb Distribution in GD14 Tissues

**Figure 8:**
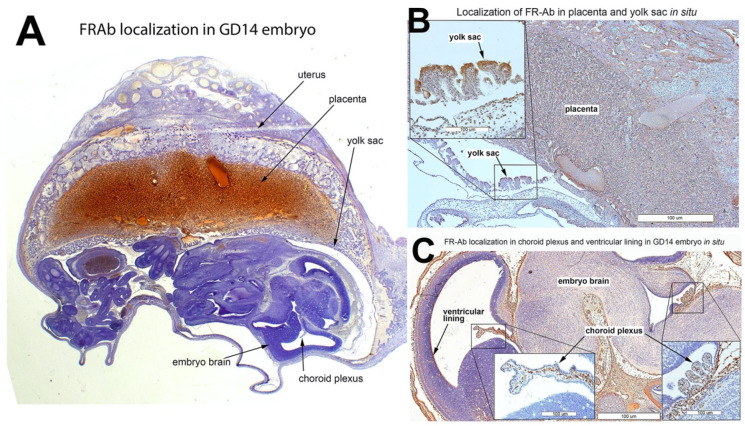
Distribution of IP-injected anti-rat FRα antibody (FRαAb) in rat placenta and embryo on GD 14; accumulation of FRαAb (brown staining) can be observed in the placenta and embryo (**A**); in the yolk sac (**B**) and in the choroid plexus, ventricular lining and in the brain tissue of the embryo (**C**). Hematoxylin (blue/purple staining) was used as a counterstain.

FRαAb administered IP accumulated extensively in the placenta (Figure 8A) and the surrounding tissues including the yolk sac (Figure 8B). In the embryo, antibody accumulation was seen in epithelial cells including the choroid plexus (Figure 8C). Such accumulation of IgG was not seen when a GD14 rat was injected with an identical dose of NR-IgG (Supplemental Data, Figure S2).

## 4. Discussion

Dietary folates derived from foods are mostly MTHF including the many reduced and oxidized polyglutamate forms. These are converted to monoglutamate by the conjugase activity in the gut prior to absorption in the upper ilium via PCFT expressed on the villi of the ileal enterocyte [6]. Cooking and processing are likely to oxidize or break down some of the folates. The breakdown products may be a source of carbon units for gut microflora to sustain growth and convert to active forms of folate. This process more than likely meets the daily dietary folate requirement under normal conditions and adequate dietary intake. The increased demand for folates during pregnancy and infancy coupled with genetic and metabolic abnormalities that affect folate pathways, require additional folate supplementation [38,39,40]. The recent identification of folate receptor autoantibodies and their significant association with NTD pregnancy, cerebral folate deficiency (CFD) syndrome, and ASD has required the use of pharmacologic doses of folinic acid to overcome the blocking of folate transport by antibodies and use alternate pathways of folate uptake to restore folate status [16,25,26,41,42]. 

Absorption and distribution of folate forms in the rat indicate that high DHFR activity exists in the rat gut with a substantial capacity to rapidly convert them to MTHF [8,43,44]. Based on the peak appearance of MTHF in the blood, at the dose administered, approximately 70% of levofolinate and d,l-folinic acid (0.6–0.7 mg) is converted to MTHF in the first hour. This drops by about 50% for folic acid, which may be attributed to the processing of folic acid through DHFR. Nevertheless, the gut appears to have a high capacity to absorb and rapidly convert to MTHF to provide the appropriate form of folate for transport out of the enterocyte via RFC expressed on the basal side. Such high DHFR activity is also present in human jejunum to convert folates to MTHF [3,8]. It is proposed that the ATP cassette MRP transporter may also transport folate [6,9]. Folic acid has low affinity for RFC and therefore, is unlikely to be transported via RFC. Since substantial folic acid is transported as such, it is likely that MRP, or some FRα that is expressed, may be carrying out this transport. However, at low doses of folic acid, most of the compound is likely to be converted to MTHF, and therefore, little or no folic acid should be appearing in circulation. The only probability in this situation is that DHFR slows substantially below the K_m_ for folic acid and therefore, some residual folic acid may remain and appears in circulation, especially with daily supplementation. Even though some recent publications have suggested an association of circulating folic acid with disorders attributed to metabolic folate deficiency, there is no evidence to corroborate an effect on folate pathways by extracellular or intracellular unmetabolized folic acid.

While folic acid can be used as a source of folate since it is readily converted to MTHF, at large doses substantial folic acid is likely to be absorbed unconverted. Even though most of the folate will be taken up by tissue cells and converted to MTHF, the excess folate in circulation will be excreted in the following 24 h or recycled via the entero-hepatic circulation [45]. Folic acid is a highly stable compound and therefore has been widely used in vitamin preparations and in food fortification. The increase in the use of vitamin supplements and consumption of fortified food has increased the folate status in the population and reduced the incidence of folate deficiency-related disorders including NTD pregnancy [40,46,47]. While there is no evidence to substantiate the claim that unmetabolized folic acid poses certain risks of interfering with folate metabolism, some recent publications have suggested its association with NTD and ASD [47,48]. Behavioral deficits in mice have been reported simply by doubling their folic acid intake [24]. A study in mice has suggested that too little or too much folic are both deleterious for brain development [19]. These observations are difficult to reconcile with dietary intake, which can vary daily, and therefore, built-in mechanisms must exist to normalize tissue distribution and utilization of nutrients. Nevertheless, providing the right form of folate in folate-dependent abnormalities would render the issues of unmetabolized folic acid inconsequential. Maternal folate requirement during pregnancy is substantially high due to the increased demand by the fetus [49]. The rapid transport and accumulation of folic acid in the placenta and distribution to the fetal tissues support the increased demand for folate during pregnancy. Incidentally, the FRαAb also shows a similar distribution profile in the placenta and embryo with blocking of folate uptake as observed with radioactive folate. However, this study also shows that at pharmacologic doses, the blocking effect of FRαAb is neutralized to provide the same amount of folate to the fetus as controls. Even though statistical differences were observed among folate forms when comparing placenta and embryo, the uptake of all forms in these tissues is more than adequate to meet the folate requirement. Selecting the correct form of folate for pharmacologic administration is critical for daily, long-term treatment. The current study suggests that Leucovorin or MTHF may be preferred when administered in larger doses. While folic acid has been used in prenatal supplements, the use of folinic acid or MTHF may be worth considering. Pharmacokinetic studies in humans have shown that MTHF is absorbed and retained better than folic acid [50]. Our rat studies also show that MTHF and both forms of folinic acid have comparable absorption. The use of Leucovorin in the treatment of ASD has shown significant improvement in language, speech and social interaction, especially more so in ASD children positive for FRαAuAb [51]. While d,l-folinic acid is a racemic mixture, the availability of levofolinate provides a metabolically active form that can be administered at half the dose of leucovorin for long-term treatment of folate receptor autoimmune disorder in ASD. Levofolinate is a relatively stable compound and could be considered for food fortification as well as a prenatal supplement.

## 5. Conclusions

During intestinal absorption, all folate forms are rapidly converted to MTHF. However, at higher doses, some unconverted folate forms appear in circulation with folic acid accounting for a larger fraction. MTHF is absorbed and transported as such into circulation. Both levofolinate and Leucovorin are more readily converted to MTHF than PGA. Based on the distribution of B-PGA and FRαAb, both appear to be rapidly distributed to the placenta and embryo. Moreover, FRαAb appears to concentrate in the placenta and consequently blocks folate transport from the dam to the fetus under a normal folate replete condition. In the presence of FRαAb, levofolinate, d,l-folinic acid, and MTHF appear to accumulate better in the placenta and fetus than folic acid when dosed orally at 4 mg/kg. Levofolinate, being more stable and fully active, could be the folate form of choice to restore fetal folate deficiency induced by FRαAb, as well as a prenatal and dietary supplement.

## Data Availability

Not applicable.

## Supplementary Materials

The following supporting information can be downloaded at: https://www.mdpi.com/article/10.3390/nu14122397/s1. Table S1: MS/MS analysis for folate forms; Figure S1: Standard curve for measuring methlyfolate in tissues; Figure S2: Specificity of staining for folate receptor antibody in the fetal brain following IP injection of normal rabbit IgG or an equivalent amount of rabbit IgG containing anti-rat folate receptor antibody.
