# Supplementary material for: Absorption and Tissue Distribution of Folate Forms in Rats: Indications for Specific Folate Form Supplementation during Pregnancy
Source: Nutrients. 2022 Jun 9;14(12):2397. doi: 10.3390/nu14122397 (PMC9228663; doi:10.3390/nu14122397)

Supplemental data

Supplemental Table 1: MS/MS analysis for folate forms.

MRM transition						
**Analyte**	**Retention time (min)**	**Q1 (*m/z*)**	**Q3 (*m/z*)**	**DP (V)**	**CE (V)**	**CXP (V)**
5-MTHF	2.3	460.3	313.0	110	23	15
Folic Acid	2.6	442	295.2	90	19	10
THF	2.3	446.3	299.0	130	23	15
Formyl-THF	2.5	474	327.0	120	24	15
$^{13}C_5$-5-MTHF	2.3	465.2	313.2	125	24	15
C13-FA	2.6	447.3	295.1	76	24	15
C13-Formyl-THF	2.5	475.2	328.1	60	26	8

Curtain gas = 20 psi, GS 1 and GS 2 = 50 psi, CAD gas = 3.5 x 10e-5 Torr.

v = volts
m/z = mass-to-charge ratio
CAD = collision-activated dissociation
psi = pound per square inch
Q1; Q3 = quadruploe 1 and 3
DP= declustering potential
CE = collision energy
CXP = collision cell exit potential
MRM transition = multiple reaction monitoring

.

Figure S1: Standard curve for measuring methylfolate in tissues.

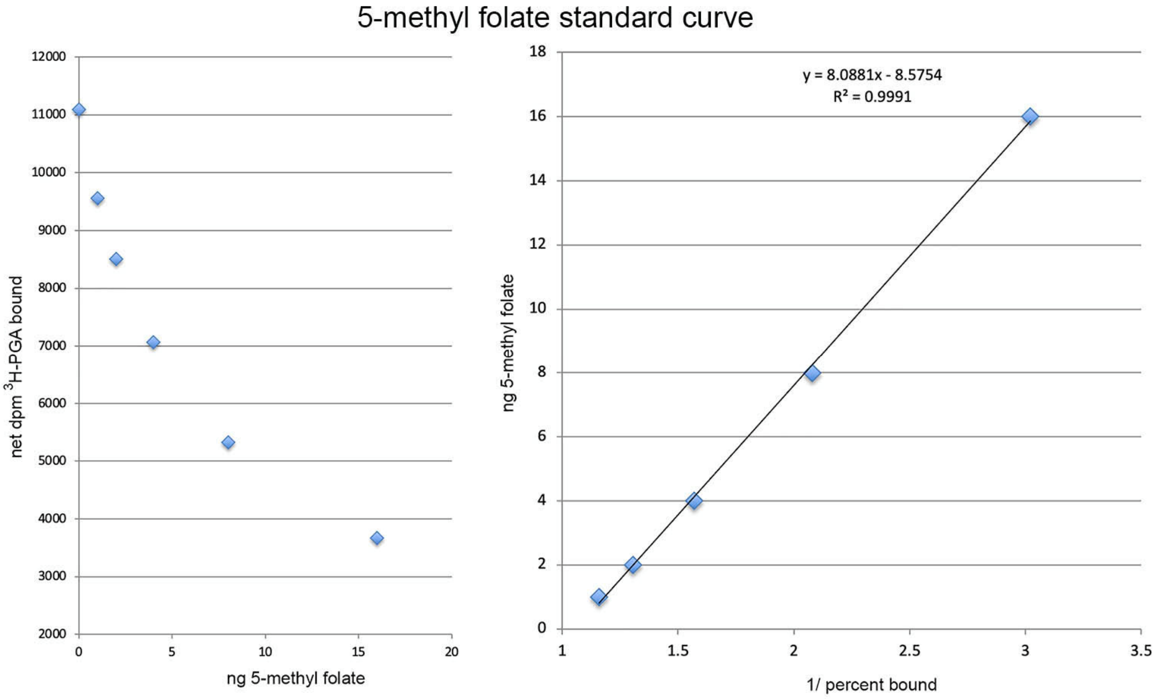

Figure S2: Specificity of staining for folate receptor antibody in the fetal brain following IP injection of normal rabbit IgG (left panel) or an equivalent amount of rabbit IgG containing anti rat folate receptor IgG (right panel). Negative staining in the left panel indicates clearance of normal rabbit IgG whereas specific staining for folate receptor antibody can be seen in the choroid plexus.

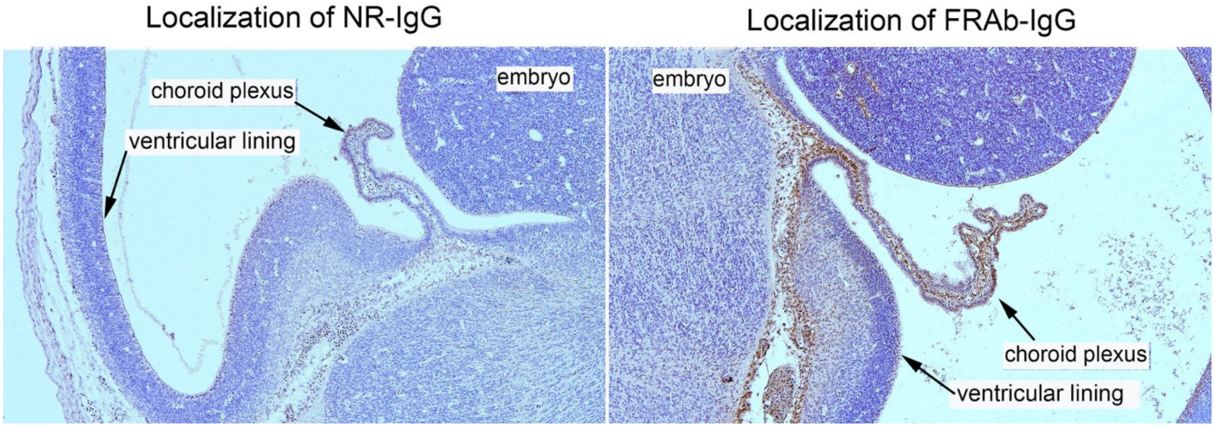

Supplement: Supplementary file 1 [file nutrients-14-02397-s001.zip › nutrients-1712353-supplementary.pdf]
